# Omicron: The pandemic propagator and lockdown instigator – what can be learnt from South Africa and such discoveries in future

**DOI:** 10.3126/nje.v11i4.41569

**Published:** 2021-12-31

**Authors:** Indrajit Banerjee, Jared Robinson, Indraneel Banerjee, Brijesh Sathian

**Affiliations:** 1-2 Sir Seewoosagur Ramgoolam Medical College, Belle Rive, Mauritius; 3 Consultant Uro oncologist and Robotic Surgeon, Apollo multi speciality Hospitals, Kolkata, West Bengal, India; 4 Geriatric and long term care Department, Rumailah Hospital, Hamad Medical Corporation, Doha, Qatar

**Keywords:** COVID-19 Virus variants, omicron SARS-CoV-2 variant, SARS-CoV-2, SARS-CoV-2 variants

## Abstract

The SARS-CoV-2 virus which causes the disease termed COVID-19 ripped through the globe in the latter part of 2019 and has left a state of fear, death and destruction in its wake. The Omicron variant was officially announced by the South African authorities on the 24th of November 2021, with the first confirmed sample of the infection being collected on the 9th of November 2021. The initial cases were flagged as a possible new variant due to the stark differences in the presentation and clinical features of the patients. At the time of Omicron’s discovery, the predominant variant circulating within South Africa was the Delta variant B.1.617.2 which typically presented with more severe and distinct symptoms.

Omicron spread rapidly within the Southern Africa and abroad, principally South Africa, Botswana, Hongkong and Israel were among the first countries to record cases of the new variant. The first European case of the Omicron variant was confirmed on the 26th of November 2021 in Belgium. Towards the end of November 2021 cases of the new variant had been confirmed and recorded in France, the United Kingdom, Germany, Portugal and Scotland. Additional cases of the Omicron variant have been confirmed in Canada, Australia, India and United States.

At this current point in the development of the Omicron upsurge in cases the international community should aim for further vaccinations among their fellow countrymen, but more so vaccine equality should be ensured. Such equality should be ensured in the developing nations as the virus does not respect any boundaries or territories and thus a higher level of vaccination worldwide will confer greater protection to the global community as a whole.

## Background

The SARS-CoV-2 virus, which causes the disease termed COVID-19 ripped through the globe in the latter part of 2019 and has left a state of fear, death and destruction in its wake. The invent of the newly found vaccines to combat the virus generated a new hope and sense of exit from the pandemic and return to normal life. International borders gradually re-opened and international travel was permitted which bolstered the kickstart of global markets and economies.

Despite the efficacy of the vaccines and their proven ability to decrease mortality and morbidity of those infected with the virus, the international community did not ensure vaccine equity and large fraction within the general populous remained strongly opposed to vaccination. The lack of international and global partnership as well as skepticism surrounding the vaccine efficacy set the stage for the development and discovery of the new variant, namely (B.1.1.529) / 21K(Omicron) which very harshly exposed the fragile and false veil of protection that the world has been living under, with weakened and eased health protocols and softer mask mandates. [1,2]

### The discovery

The Omicron variant was officially announced by the South African authorities on the 24th of November 2021, with the first confirmed sample of the infection being collected on the 9th of November 2021. The initial cases were flagged as a possible new variant due to the absence of anosmia and dysgeusia in the clinical presentation of the patients. At the time of Omicron’s discovery, the predominant variant circulating within South Africa was the Delta variant B.1.617.2 which typically presented with more severe and stark symptoms. The cases of this suspected new variant were flagged due to their mildness and very docile disease progression. Subsequent to the suspicions of the circulation of a new variant, genome sequencing was performed and the new variant was discovered. The initial findings of the mutations present in Omicron called for the WHO to label the variant as one of “concern”, due to it having more mutations than the Delta variant which is still causing thousands of deaths internationally. [3-5]

### The mutations

The greatest concern caused by the newly discovered Omicron variant is the myriad of mutations it possesses.The omicron variant is shown to have over 50 mutations with more than 30 of them explicitly being in the spike protein of the virus. The concern about the mutations within the spike protein is the fact that most of the vaccines currently primarily target the spike proteins of the virus for their mechanism of action, and thus such mutations raise the concern about vaccine efficacy against this new variant, as the possibility of existing vaccines not being effective or offering adequate protection against the infection effectively places the globe back to the early days in 2019 and 2021 when the virus was first discovered. An analysis of more than 130 Omicron variant genetic sequences between November 9th and 28th have depicted that the Omicron variant has subdivided and diverged into a minimal of 6 major subgroups with variations in the amino acid 214.

Another matter for concern for virologists and epidemiologists was the fact that some of the mutations were completely novel and their function and or effect on the human body is completely unknown. [3,6,7]

### The spread

South Africa, Botswana, Hongkong and Israel were among the first countries to record cases of the new variant and the Omicron variant spread rapidly within the Southern African countries. The first European case of the Omicron variant was confirmed on the 26th of November 2021 in Belgium. Towards the end of November 2021 cases of the new variant had been confirmed and recorded in France, the United Kingdom, Germany, Portugal and Scotland. Additional cases of the Omicron variant have been confirmed in Canada and Australia,India and United States. [8] Omicron became the most common variant of newly diagnosed Corona virus infection in United States (73%) by December 20^th^, 2021.[9]

The small coastal island of Mauritius closed its boarders to South Africa from the 29th of November 2021, it however recorded its first local case from South Africa in the West of the island on Friday the 10th of November 2021. [10]

India recorded its first two cases of Omicron in a 66-year-old South African tourist and a 46-year-old native doctor in Bengaluru who had no travel history. The Indian authorities reported that both of the cases suffered from mild symptoms. It was reported that contact tracing was undertaken and all the 24 primary and 240 secondary contacts of the above two patients were negative. As of 1st of December, 2021, India declared new travel restrictions from passengers arriving from high risk countries namely being the United Kingdom, South Africa, Bangladesh, Hong Kong, Israel and New Zealand. [11] The total number of Omicron positive cases in India till date is 781 and India had also reported the first Omicron related death. [12]

### The clinical features

With the limited available data the initial reported symptoms and signs of patients presenting with the omicron variants is milder than that of the Delta variant. More docile symptoms such as headache, slight fever, rhinorrhoea and sore throat resembling the common cold have been reported. It is also speculated that the Omicron variant is more transmissible than Delta. As further studies are underway more data and symptomatology of the virus will be brought to light.[13,14]

### The vaccination and booster debarkle

The discovery of Omicron has further catapulted the immunization and booster dosing schedule debarked globally, with wide arrays of views, opinions and movements. The increase in daily numbers of positive cases with the new variant in countries like Israel and the United Kingdom (UK) have proven that even with high booster dose immunizations, the development of new variants such as Omicron still pose a threat due to the plasticity of the mutations of the virus. Factions within the scientific community are calling for vaccine equaity and it is hypothesized that by raising the immunization levels in developing countries,the developments of further mutations will be circumvented and thus will nullify the need for such rigid and frequent booster dose campaigns. Such equaity should be ensured in the developing nations as the virus does not respect any boundaries or territories and thus a higher level of vaccination worldwide will confer greater protection to the global community as a whole. [15]

### The reaction

The aggressive and rapid reaction by countries, the global stock market and people alike exposed the real danger that this viral entity still poses to the global community at large on a psychosocial, educational, diplomatic and institutional basis. Juxtaposed to the sheer danger of the development of such variants and the threats they pose, the action and swift travel bans put into place raise further concerns for global partnership and development. The United Kingdom were the first country to place travel bans on flights into the Southern African continents, which set into motion a sequence of events which lead to global bans on air travel within the region. Such bans were criticized and concerns were raised that it will discourage other countries to report such discoveries in the future. The travel ban from the Southern African continents to the United Kingdom was officially lifted on the 15th of December 2021 and Omicron variant became the dominant strain in UK by December 15^th^, 2021. [16] The current research and findings are hopeful and report that the Pfizer vaccine will be effective against the new variant, and reports from South Africa record milder symptoms with lower hospitalization rates being observed with individuals infected with the new variant. [17-19]

### Recommendations on mask and PPE

The WHO (World Health Organization) has released an updated mandate as well as recommendations in light of the development of the Omicron variant. The WHO recommendations outline the specific use of high-level respirators and not simple masks. Respirators rated to the level of FFP2, FFP3, N95 or higher-level are recommended. In addition to the use of the above specific respirators it has been stated that various other PPE (personal protective equipment) as well as specific COVID conduct and hand hygiene should be maintained. The specific use of such equipment has been mandated specifically due to the increased transmissibility of the new Omicron variant. In addition to the above guidelines the WHO has also released an IPC (infection prevention and control) stratagem for hospital administrators which highlights the areas of focus to best combat and protect both hospital staff and patients whilst minimizing the cross contamination in areas with COVID-19 outbreaks. The document outlines the safe management of the flow of patients and staff, the safe evaluation of the care environment and remediation in such cases where improvement is needed, the optimal use and application of the PPE (personal protective equipment) equipment as well as the concept of universal masking, increased strengthening of screening, testing and physicals distancing, vaccination in health workers as well as the ongoing training and infection prevention and control. [20,21]
